# Supplementary material for: In vivo gene expression profile of Haemophilus influenzae during human pneumonia
Source: Microbiol Spectr. 2023 Sep 14;11(5):e01639-23. doi: 10.1128/spectrum.01639-23 (PMC10581191; doi:10.1128/spectrum.01639-23)
Supplement: Supplemental file 1: R scripts — R scripts used for differential gene expression analysis and figures [file spectrum.01639-23-s0001.docx]

***In vivo* gene expression profile of *Haemophilus influenzae* during human pneumonia**

Linnea Polland^1,2^, Hanna Rydén^1,2^, Yi Su^1^, Magnus Paulsson^1,2 #^

Author affiliations

1. Infection medicine, Department of Clinical Sciences Lund, Medical Faculty, Lund University, Lund, Sweden
2. Clinical Microbiology, Office for Medical Services, Region Skåne, Lund, Sweden

**Supplemental file 1: R scripts**

#####################################################################

### IMPORT READS FROM KALLISTO HI ###

#####################################################################

library(readxl)

library(tools)

library(readr)

library(tximport)

library(DESeq2)

dir <- "...kallisto"

file_names <- list.files(dir)

files <- file.path("kallisto", file_names, "3_kallisto", "HI_core", "abundance.tsv") #HI_pan for pan-analyses

samples <- read_excel("samples.xlsx")

names(files) <- samples$sample

list(files)

tx2gene <- read.delim("tx2gene_core.txt",header=F)

head(tx2gene)

txi <- tximport(files, type = "kallisto", tx2gene = tx2gene)

names(txi)

head(txi$counts)

#####################################################################

### DESEQ HI ###

#####################################################################

library(DESeq2)

library(ggrepel)

library(vsn)

sample_info <- samples

rownames(sample_info) <- samples$file

sample_info

colData<- sample_info[,c("condition","subject", "qc", "run", "sample")]

colData$condition <- factor(colData$condition)

colData$subject <- factor(colData$subject)

colData$qc <- factor(colData$qc)

colData$run <- factor(colData$run)

colData$sample <- factor(colData$sample)

##Import dataset from kallisto (dds_p if pan):

dds <- DESeqDataSetFromTximport(txi, colData = colData, design = ~ subject + condition)

##Remove low qc:

dds<- dds[, !colData$qc == 2]

dds$subject <- droplevels(dds$subject)

##Collapse replicates from different runs:

dds <- collapseReplicates(dds, dds$sample, renameCols = TRUE)

##Compare in vivo to in vitro:

dds$condition <- relevel(dds$condition, ref = "In vitro")

##Filter low count reads:

keep <- rowSums(counts(dds)) >= 10

dds <- dds[keep,]

dds_all <- DESeq(dds)

res_all <- results(dds_all)

head(res_all[order(res_all$padj),])

sum(res_all$padj < 0.1, na.rm=TRUE)

##Top DEGs (padj <= 0.05):

res05 <- results(dds_all, alpha=0.05)

summary(res05)

sig_genes <- (res_all[(res_all$padj<=0.05 & abs(res_all$log2FoldChange) >=1), ])

summary(sig_genes)

write.table(sig_genes, paste0('DEG_p_adj_0.05_logFC_1.txt'), col.names = T, row.names = T, sep = '\t', quote = F)

write.table(results(dds_all), paste0('DEG_all_logFC_core.txt'), col.names = T, row.names = T, sep = '\t', quote = F)

##Log fold change shrinkage:

library(apeglm)

resultsNames(dds_all)

resLFC <- lfcShrink(dds_all, coef="condition_In.vivo_vs_In.vitro", type="apeglm")

resLFC

##Variance:

library("vsn")

vsd <- vst(dds_all, blind=FALSE)

rld <- rlog(dds_all, blind=FALSE)

head(assay(vsd), 3)

ntd <- normTransform(dds_all)

meanSdPlot(assay(ntd))

meanSdPlot(assay(rld))

meanSdPlot(assay(dds_all))

#####################################################################

### PCA PLOT ###

#####################################################################

library(ggplot2)

library(RColorBrewer)

vst <- varianceStabilizingTransformation(dds, blind = F)

data_id <- plotPCA(vst, ntop=1100, returnData=T, intgroup=c("condition","subject"))

plotPCA(vst, ntop=1100, returnData=F, intgroup=c("condition","subject")) #to get percentage

ann_colors_point_alt = c(LUIN_26 = "#9e0142", LUIN_28 = "#d53e4f", LUIN_29 = "#F46D43", LUIN_31 = "#fdae61",

LUIN_33 = "#fee08b", MAAK_03 = "#abdda4", MAIV_24 = "#66c2a5", MAIV_32 = "#3288bd",

MAIV_34 = "#5E4FA2", NTHi_3655 = "#ACA8B5")

ggplot(data=data_id) +

geom_point(aes(x=PC1, y=PC2, color=subject, shape=condition), size=2.5) +

scale_color_manual(values = ann_colors_point_alt)

#####################################################################

### HEATMAPS ###

#####################################################################

library(pheatmap)

library(ggplot2)

library(RColorBrewer)

##Top30 logFC heatmap, with row centering:

select1 <- order(res_all$log2FoldChange,decreasing=T)[1:14]

select2 <- order(res_all$log2FoldChange,decreasing=F)[1:16]

select_all <- c(select1,select2)

df <- as.data.frame(colData(dds_all)[,c("condition","subject")])

zztop<-assay(vsd)[select_all,]

zztop<- zztop-rowMeans(zztop)

ann_colors_heat_alt = list(

"condition" = c("In vitro" = "#B6DDE3", "In vivo" = "#f57c73"),

"subject" = c(LUIN_26 = "#9e0142", LUIN_28 = "#d53e4f", LUIN_29 = "#F46D43", LUIN_31 = "#fdae61",

LUIN_33 = "#fee08b", MAAK_03 = "#abdda4", MAIV_24 = "#66c2a5", MAIV_32 = "#3288bd",

MAIV_34 = "#5E4FA2", NTHi_3655 = "#ACA8B5"))

pheatmap(zztop, cluster_rows=T, show_rownames=TRUE,breaks=seq(-3,3,length.out=101),

color = colorRampPalette(rev(brewer.pal(n = 10, name =

"RdYlBu")))(100), cluster_cols=FALSE, annotation_col=df,

annotation_colors = ann_colors_heat_alt,

fontfamily = "serif",

filename="heatmap_FC_top30.pdf")

##Heatmap of top 250 variable with cluster_cols and row centering:

select <- assay(vsd) ##get the data

select<- apply(select,1,var) ##calculate var of each gene

select<- select > quantile(select, 1-(250/length(select)) ) #top 250 genes

df <- as.data.frame(colData(dds_all)[,c("condition","subject")])

zztop<-assay(vsd)[select,]

zztop<- zztop-rowMeans(zztop)

pheatmap(zztop, cluster_rows=T, show_rownames=F,breaks=seq(-3,3,length.out=101),

color = colorRampPalette(rev(brewer.pal(n = 10, name =

"RdYlBu")))(100), cluster_cols=T, annotation_col=df,

treeheight_row = 0,

annotation_colors = ann_colors_heat_alt,

clustering_distance_rows = "correlation", #=pearson

clustering_distance_cols = "correlation",

clustering_method = "ward.D",

fontfamily = "serif",

filename="heatmap_FC_core.pdf")

#####################################################################

### PLOT COUNTS of top30 ###

#####################################################################

library(tidyverse)

library(tidyr)

library(ggplot2)

dds_sf<-estimateSizeFactors(

dds,

type = c("ratio", "poscounts", "iterate"),

locfunc = stats::median,

quiet = FALSE)

normalized_counts <- counts(dds_sf, normalized=TRUE)

head(normalized_counts)

normalized_counts_log<-(normalized_counts)

normalized_counts_log[normalized_counts_log == 0] <- 1

normalized_counts_log<-log2(normalized_counts_log)

norm_counts<-as_tibble(normalized_counts_log, rownames = "gene")

res_all_tb<-as_tibble(res_all, rownames = "gene")

top_n <- res_all_tb %>%

arrange( -abs(log2FoldChange) ) %>% #Arrange rows by absolute logFC values

pull(gene) %>% #Extract character vector of ordered genes

head(n=30) #Extract the top 30 genes

top_n_norm <- norm_counts %>%

filter(gene %in% top_n)

gathered_top <- top_n_norm %>%

gather(colnames(top_n_norm)[2:23], key = "samplename", value = "normalized_counts")

stype<-gathered_top$samplename %>% #select(samplename) %>%

as.vector() %>%

str_replace_all("^HI.*","in vitro") %>%

str_replace_all("^LU.*","in vivo") %>%

str_replace_all("^MA.*","in vivo") %>%

as_tibble()

gathered_top2 <- bind_cols(gathered_top,sampletype=stype)

COLS = c("#B6DDE3","#F57C73")

ggplot(gathered_top2) +

geom_point(aes(x = gene, y = normalized_counts, color = value, shape = value), size = 2) +

scale_color_manual(values = COLS) +

scale_shape_manual(values = c(16,18)) +

geom_segment(

data = medians,

mapping = aes(x = xmin, xend = xmax, y = y, yend = y,color = value), linewidth=0.8) +

xlab("") +

ylab("log2(Normalized Counts)") +

ggtitle("Expression of top 30 DEGs") +

theme_minimal() +

theme(axis.text.x = element_text(angle = 45, hjust = 1, color = "black")) +

theme(plot.title = element_text(hjust = 0.5),

text = element_text(family = "serif"),

legend.title=element_blank(),

legend.text = element_text(size = 10))

#####################################################################

### ZEROS IN CORE ###

#####################################################################

library(proxyC)

table(rowZeros(counts(dds)))

counts(dds)["zwf",]

which( rowSums(counts(dds)==0)==7)

#####################################################################

### VOLCANO PLOT ###

#####################################################################library(EnhancedVolcano)

library(EnhancedVolcano)

vp<-plot(EnhancedVolcano(res_all,

lab = rownames(res_all),

x = 'log2FoldChange',

y = 'pvalue',

subtitle = "",

caption = "Total no. of variables = 1067",

legendDropLevels = T,

legendLabels = c("NS","Log2 FC >= 1","P-value <= 0.05","DEGs"),

col = c("#A5C3CE","#66c2a5","#3288bd","#9e0142"),

colAlpha = 0.50,

title = "",

pCutoff = 0.05,

FCcutoff = 1,

pointSize = 1.0,

labSize = 5.0,

xlim = c(-6, 6),

ylim = c(0, 20)))

sapply(vp$layers, function(x) class(x$geom)[1])

vp$layers[[4]]$aes_params$family <- "serif"

vp <- vp +

theme(text = element_text(family = "serif"))

pdf(file = "volcano_plot.pdf", width = 10, height = 6)

vp

dev.off()

#####################################################################

### GO ANALYSES AND TABLES: TopGo ###

#####################################################################

library(topGO)

library(readxl)

library(stringr)

library(dplyr)

###Make list of gene to GO-id (http://127.0.0.1:14779/library/topGO/doc/topGO.pdf):

HI<-read_excel("DEGs n=325.xlsx")

HI2<-read_excel("Non-DEGs n=736.xlsx")

HI<-HI[c("Mapped ID's","GO database BP complete")]

HI2<-HI2[c("Mapped ID's","GO database BP complete")]

HI3<-rbind(HI,HI2)

summary(HI3)

newList<-str_split(unlist(HI3[,2]),";")

newList<-lapply(newList,function(x) str_extract(x,pattern="GO:\\d*") )

names(newList)<-unlist(HI3[,1])

head(newList)

length(newList)

##Fetch list of interesting up- and downregulated genes

##(Excel-files of GO annotation in which up and down-regulated DEGs are split):

sign_up<-read_excel("Sign up BP.xlsx")

sign_down<-read_excel("Sign down BP.xlsx")

##Define universe of gene symbols/annotation

geneList<-names(newList)

##Define subset of genes in "geneList" that are interesting (DEGs):

genes_up <- sign_up$`Mapped ID's`

genes_down <- sign_down$`Mapped ID's`

##Define geneList as a factor where signDiff is koded

geneList_up<-as.factor( as.integer( geneList %in% genes_up ) )

names(geneList_up)<-names(newList)

table(geneList_up)

geneList_down<-as.factor( as.integer( geneList %in% genes_down ) )

names(geneList_down)<-names(newList)

table(geneList_down)

##Create new topGO data object (GOdata object):

GOdata_up <- new("topGOdata", ontology = "BP", allGenes = geneList_up,

annot = annFUN.gene2GO, gene2GO = newList)

GOdata_down <- new("topGOdata", ontology = "BP", allGenes = geneList_down,

annot = annFUN.gene2GO, gene2GO = newList)

##Perform statistics on topGO data objects:

resultFis2_up <- runTest(GOdata_up, algorithm = "weight01", statistic = "fisher")

resultFis2_up

resultFis2_down <- runTest(GOdata_down, algorithm = "weight01", statistic = "fisher")

resultFis2_down

##Create GenTables of result:

GenTable1<-GenTable(GOdata_up, classicFisher = resultFis2_up,

orderBy = "resultFis2_up", ranksOf = "classicFisher",

numChar = 99,

topNodes = "50")

GenTable2<-GenTable(GOdata_down, classicFisher = resultFis2_down,

orderBy = "resultFis2_down", ranksOf = "classicFisher",

numChar = 99,

topNodes = "50")

##Modify (optional):

GenTable1$classicFisher <- as.numeric(GenTable1$classicFisher)

GenTable1 <- GenTable1[GenTable1$classicFisher < 0.05,] # filter terms for p<0.5

GenTable1$'Fold enrichment'<-(GenTable1$Significant/GenTable1$Expected)

GenTable1

GenTable2$classicFisher <- as.numeric(GenTable2$classicFisher)

GenTable2 <- GenTable2[GenTable2$classicFisher < 0.05,] # filter terms for p<0.5

GenTable2 <- GenTable2 %>% relocate('Fold enrichment', .before = 'classicFisher')

GenTable2

##Modify headers:

GenTable1<-rename(GenTable1,"Gene ontology ID"="GO.ID",

"Fisher's exact test"="classicFisher",

"Annotated (n)"="Annotated",

"Significant (n)"="Significant",

"Expected (n)"="Expected")

GenTable2<-rename(GenTable2,"Gene ontology ID"="GO.ID",

"Fisher's exact test"="classicFisher",

"Annotated (n)"="Annotated",

"Significant (n)"="Significant",

"Expected (n)"="Expected")

##Save as a pdf table:

library(dplyr)

library(kableExtra)

library(magick)

library(webshot)

library(animation)

GenTable1 %>%

kbl(caption = "Biological Processes more common in vivo",booktabs = T) %>%

kable_classic(full_width = F, html_font = "times new roman") %>%

kable_styling(font_size=11,htmltable_class="lightable-classic") %>%

save_kable(paste0(path,"/topGOterms_up_tnr_top50.html"))

webshot("topGOterms_up_tnr_top50.html", "topGOterms_up_tnr_top50.pdf")

GenTable2 %>%

kbl(caption = "Biological processes less common in vivo",booktabs = T) %>%

kable_classic(full_width = F, html_font = "times new roman") %>%

kable_styling(font_size=11,htmltable_class="lightable-classic") %>%

save_kable(paste0(path,"/topGOterms_down_tnr_top50.html"))

webshot("topGOterms_down_tnr_top50.html", "topGOterms_down_tnr_top50.pdf")

##Finding annotated genes of specific GO terms:

num.ann.genes <- countGenesInTerm(GOdata,whichGO ="GO:0006826") ## the number of annotated genes

num.ann.genes

ann.genes <- genesInTerm(GOdata,whichGO ="GO:0006094") ## get list of the annotated genes

ann.genes

#####################################################################

##BOXPLOT OF NORMALIZED COUNTS:genes of sign GO terms##

#####################################################################

library(reshape2)

library(readxl)

library(ggplot2)

library(dplyr)

library(RColorBrewer)

library(cowplot)

##Genes of IMP/GO:0006189 (same basic code used for TCA [GO:0006108, GO:0006106 and GO:0006099] and ##iron [GO:0006880 and GO:0006826 ]related genes):

purB<-counts(dds_sf["purB",],normalized=T)

purC<-counts(dds_sf["purC",],normalized=T)

purD<-counts(dds_sf["purD",],normalized=T)

purE<-counts(dds_sf["purE",],normalized=T)

purF<-counts(dds_sf["purF",],normalized=T)

purH<-counts(dds_sf["purH",],normalized=T)

purK<-counts(dds_sf["purK",],normalized=T)

purL<-counts(dds_sf["purL",],normalized=T)

IMP<-rbind(purB,purC,purD,purE,purF,purH,purK,purL)

##Make csv file with values:

IMP<-as.data.frame(IMP)

IMP<-t(IMP)

write.csv(IMP,file=paste0(path,"/IMP.csv"),quote=F)

##Then copy paste into excel xlsx file where in vitro/in vivo is specified, then fetch it again:

IMP_plot<-read_excel("IMP.xlsx")

IMP_plot$purB<-log2( as.numeric(IMP_plot$purB) +1)

IMP_plot$purC<-log2( as.numeric(IMP_plot$purC) +1)

IMP_plot$purD<-log2( as.numeric(IMP_plot$purD) +1)

IMP_plot$purE<-log2( as.numeric(IMP_plot$purE) +1)

IMP_plot$purF<-log2( as.numeric(IMP_plot$purF) +1)

IMP_plot$purH<-log2( as.numeric(IMP_plot$purH) +1)

IMP_plot$purK<-log2( as.numeric(IMP_plot$purK) +1)

IMP_plot$purL<-log2( as.numeric(IMP_plot$purL) +1)

IMP_plot<-rename(IMP_plot, "purB*" = "purB","purD*" = "purD","purE*" = "purE","purF*" = "purF","purH*" = "purH","purK*" = "purK","purL*" = "purL")

##Modify data:

IMP_box<-cbind(type= rep(IMP_plot$Sampletype,8),gene=rep(colnames(IMP_plot)[-1:-2],each=22),value=unlist(IMP_plot[,-1:-2]))

IMP_box<-as_tibble(IMP_box)

IMP_box$value<-as.numeric(IMP_box$value)

##Combination boxplots:

COLS = c("#B6DDE3","#F57C73")

avalues= c(0.65, 0.95)

fillCOLS = sapply(1:2,function(i)alpha(COLS[i],avalues[i]))

IMP_bp <- ggplot(IMP_box, aes(x=gene,y=value,fill=type)) +

geom_boxplot() +

scale_fill_manual(values = fillCOLS) +

xlab("") +

ylab("log2(Normalized counts +1)") +

ggtitle("Genes involved in 'de novo' IMP biosynthetic process") +

labs(fill = "") +

guides(fill = "none") + #remove legend

theme_minimal() +

theme(axis.text.x = element_text(size = 12, color = "black"),

axis.text.y = element_text(size = 12),

axis.title.y = element_text(size = 12),

plot.title = element_text(size = 14),

text = element_text(family = "serif"))

iron_bp <- ggplot(iron_box, aes(x=gene,y=value,fill=type)) +

geom_boxplot() +

scale_fill_manual(values = fillCOLS) +

#scale_fill_brewer(palette = "Pastel2") #alternative colors

xlab("") +

ylab("log2(Normalized counts +1)") +

ggtitle("Genes involved in sequestering and transport of iron") +

labs(fill = "") +

guides(fill = "none") + #remove legend

theme_minimal() +

theme(axis.text.x = element_text(size = 12, color = "black"),

axis.text.y = element_text(size = 12),

axis.title.y = element_text(size = 12),

plot.title = element_text(size = 14),

TCA_bp<-ggplot(TCA_box, aes(x=gene,y=value,fill=type)) +

geom_boxplot(width = 0.5) +

scale_fill_manual(values = fillCOLS) +

xlab("") +

ylab("log2(Normalized counts +1)") +

ggtitle("Genes involved in the tricarboxylic acid cycle") +

labs(fill = "") +

theme_minimal() +

theme(axis.text.x = element_text(size = 12, color = "black"),

axis.text.y = element_text(size = 12),

axis.title.y = element_text(size = 12),

plot.title = element_text(size = 14),

legend.text = element_text(size = 14),

legend.key.size = unit(1, "cm"),

text = element_text(family = "serif"))

third_plot<-plot_grid(iron_bp, IMP_bp, labels = c("B", "C"))

plot_grid(TCA_bp, third_plot,

nrow = 2,

labels = c("A",""))
