# Supplementary material for: In vivo gene expression profile of Haemophilus influenzae during human pneumonia
Source: Microbiol Spectr. 2023 Sep 14;11(5):e01639-23. doi: 10.1128/spectrum.01639-23 (PMC10581191; doi:10.1128/spectrum.01639-23)
Supplement: Supplemental Tables S1-5 and Figure S1-3 — Supplemental tables about genomes used for pan genome creation, clinical data about study subjects, basic sequencing data and GO-term analysis. Supplemental figures about the pan genome creation and differential gene expression using the pan genome as reference. [file spectrum.01639-23-s0002.docx]

***In vivo* gene expression profile of *Haemophilus influenzae* during human pneumonia**

Linnea Polland^1,2^, Hanna Rydén^1,2^, Yi Su^1^, Magnus Paulsson^1,2 #^

Author affiliations

1. Infection medicine, Department of Clinical Sciences Lund, Medical Faculty, Lund University, Lund, Sweden
2. Clinical Microbiology, Office for Medical Services, Region Skåne, Lund, Sweden

**Supplemental Tables S1-5 and Figure S1-3**

Supplemental Table S1

| Accession number | Strain name | Serotype | MLST |
| --- | --- | --- | --- |
| ASM2730v1 | H. influenzae Rd KW20 | NTHi | 1621 |
| ASM1218v1 | H. influenzae 86-028NP | NTHi | 33 |
| ASM1646v1 | H. influenzae PittEE | NTHi | 107 |
| ASM1648v1 | H. influenzae PittGG | NTHi | 2394 |
| ASM19787v1 | H. influenzae F3031 | NTHi | 65 |
| ASM20047v1 | H. influenzae F3047 | NTHi | 70 |
| ASM21087v1 | H. influenzae 10810 | b | 6 |
| ASM16557v1 | H. influenzae R2846 | NTHi | 1622 |
| ASM16552v1 | H. influenzae R2866 | NTHi | 99 |
| ASM46525v1 | H. influenzae KR494 | f | 124 |
| ASM69836v1 | H. influenzae CGSHiCZ412602 | NTHi | 712 |
| ASM76707v1 | H. influenzae Hi375 | NTHi | 3 |
| ASM93157v1 | H. influenzae 477 | NTHi | 1 |
| ASM93160v1 | H. influenzae C486 | NTHi | 244 |
| ASM93162v1 | H. influenzae 723 | NTHi | 14 |

LEGEND SUPPLEMENTAL TABLE S1: List of the 15 reference strains used for creation of core and pan genome, their ASM accession number, serotype, and Multi Locus Sequence Type (MLST).

Supplemental Table S2

| *Study ID* | LUIN_26 | LUIN_28 | LUIN_29 | LUIN_31 | LUIN_33 | MAAK_03 | MAIV_24 | MAIV_32 | MAIV_34 |
| --- | --- | --- | --- | --- | --- | --- | --- | --- | --- |
| *Age* | 79 | 38 | 77 | 71 | 68 | 70 | 63 | 69 | 76 |
| *Sex* | Woman | Woman | Man | Woman | Man | Man | Woman | Man | Man |
| *Smoking habits* | Never smoker | Never smoker | Never smoker | Prev. smoker | Prev. smoker | Prev. smoker | Smoker | Smoker | Smoker |
| *Previous medical conditions and comorbidities* |  |  |  |  |  |  |  |  |  |
| *COPD* | No | No | No | Yes | No | No | Yes | Yes | Yes |
| *Asthma* | No | No | No | Yes | Yes | No | No | No | No |
| *Emphysema* | Yes | No | No | No | No | No | No | No | No |
| *Diabetes mellitus* | No | No | Yes | No | Yes | No | No | No | No |
| *Cardiovascular disease* | No | No | Yes | No | No | Yes | No | Yes | Yes |
| *Immunodeficiency* | No | No | No | Yes | Yes | No | No | No | No |
| *Ongoing treatment at study enrollment* |  |  |  |  |  |  |  |  |  |
| *Immunosuppressive treatment, (syst.=systemic, inh.=inhalation)* | No | No | No | Yes (inh.) | Yes (syst.) | No | No | Yes (syst.) | No |
| *Heparin or LMHW* | No | No | No | No | No | No | Yes | Yes | Yes |
| *Antibiotic treatment last 5 days* | No | Yes, DOX | No | Yes, DOX | No | Yes, PHE | No | No | No |
| *Clinical data at enrollment* |  |  |  |  |  |  |  |  |  |
| *Purulent sputum* | Yes | Yes | Yes | Yes | Yes | NA | Yes | NA | No |
| *Temp > 38C or < 35C last 24 h* | Yes | No | No | Yes | NA | Yes | Yes | Yes | No |
| *New pulmonary infiltrate* | Yes | Yes | Yes | Yes | Yes | No | Yes | Yes | NA |
| *Pneumonia-symptom duration (days)* | 2 | 7 | 2 | 1 | 11 | 3 | 2 | 1 | 0 |
| *NEWS score* | 5-6 p | NA | ≥ 7 p | 1-4 p | 5-6 p | 1-4 p | ≥ 7 p | ≥ 7 p | NA |
| *CRB-65 total points* | 1 | 0 | 1 | 1 | 1 | 2 | 2 | 3 | 1 |
| *qSOFA total points* | 0 | 0 | 1 | 0 | 1 | 1 | 1 | 3 | NA |
| *Laboratory results* |  |  |  |  |  |  |  |  |  |
| *Plasma-CRP (mg/l)* | 317 | 68 | 365 | 66 | 140 | 325 | 252 | 70 | 293 |
| *White blood cell count (10^9^/l)* | 17.1 | 15.6 | 11.4 | 16.5 | 13.2 | 15.8 | 10 | 18.1 | 7.4 |
| *Bacteria in sample other than H. influenzae* | No | No | No | No | M.cat | No | Pnc, S.aureus | No | Pnc, K.aer, C.alb, mxfl |
| *Management and prescriptions* |  |  |  |  |  |  |  |  |  |
| *Antibiotic treatment received in the ED* | Yes, CLI | Yes, AMO | Yes, BEN | Yes, PIT | Yes, PIT | Yes, BEN | NA | Yes, PIT | No |
| *Admitted to hospital ward* | Yes | No | Yes | Yes | Yes | Yes | Yes, ICU | Yes, ICU | Yes, ICU |
| *Type of antibiotic in ward* | DOX | NA | BEN | PIT | PIT | BEN | CTA | PIT | CTA |
| *SIR^1^-classification for given antibiotic* | S | S | R | S | S | S | S |  | S |
| *Endotracheal intubation and mechanical ventilation* | No | NA | No | No | No | No | Yes | Yes | Yes |
| *FiO2* | NA | NA | NA | NA | NA | NA | 30 | 70 | 60 |
| *Days of ventilator treatment* | NA | NA | NA | NA | NA | NA | 1 | 1 | 2 |

LEGEND SUPPLEMENTAL TABLE S2: Demographic and clinical data for all included study subjects. Comorbidities, previous and ongoing medications, vital data, laboratory values were retrieved from the hospital records at the emergency departments (ED) or intensive care units (ICU), and scores (NEWS, CRB-65 and qSOFA) were calculated from these values. Three patients were treated with antibiotics at enrollment and all patients were treated with antibiotics after sampling. These antibiotic drugs were used, as indicated in the table: amoxicillin (AMO), benzylpenicillin (BEN), cefotaxime (CTA), clindamycin (CLI), doxycycline (DOX), phenoxymethylpenicillin (PHE) and piperacillin-tazobactam (PIT). The culture results indicated that three samples contained additional microbial species to *Haemophilus influenzae*, including *Moraxella catarrhalis* (M.cat), *Streptococcus pneumoniae* (Pnc), *Staphylococcus aureus* (S.aureus), *Klebsiella aerogenes* (K.aer), *Candida albicans* (C.alb), and a mixed microbial flora (mxfl). No study subject suffered from cystic fibrosis, bronchiechtasis, pulmonary fibrosis, pulmonary cancer or other tumor disease,. ^1^SIR-classifications: S: Susceptible, Standard dosing regimen, I: Susceptible, Increased exposure, R: Resistant according to the EUCAST guidelines. Missing data is indicated as NA.

Supplemental Table S3

| File Name | M Seqs | Dups (%) | GC (%) | rRNA (%) | Length | Seq. run | Accession number |
| --- | --- | --- | --- | --- | --- | --- | --- |
| LUIN_26_S2_R1_001 | 233.8 | 92.6 | 52.0 | 33.6 | 76 bp | 2020_185 | ERR11532861 |
| LUIN_26_S4_R1_001 | 233.9 | 93.8 | 51.0 | 59.4 | 90 bp | 2021_145 | ERR11532895 |
| LUIN_26_S5_R1_001 | 9.6 | 71.5 | 52.0 | 30.3 | 72 bp | 2020_143 | ERR11532918 |
| LUIN_26_S5_R2_001 | 9.6 | 69.9 | 54.0 | 30.3 | 73 bp | 2020_143 | ERR11532918 |
| LUIN_29_S5_R1_001 | 215.2 | 86.6 | 48.0 | 16.7 | 95 bp | 2021_145 | ERR11532904 |
| LUIN_31_S6_R1_001 | 249 | 84.4 | 43.0 | 5.7 | 97 bp | 2021_145 | ERR11532911 |
| LUIN_33_S4_R1_001 | 282.3 | 90.2 | 48.0 | 35.1 | 90 bp | 2020_185 | ERR11532919 |
| LUIN_33_S7_R1_001 | 11.1 | 62.0 | 49.0 | 33.9 | 88 bp | 2020_143 | ERR11532920 |
| LUIN_33_S7_R2_001 | 11.1 | 58.4 | 50.0 | 33.9 | 88 bp | 2020_143 | ERR11532920 |
| MAAK_03_S7_R1_001 | 264.7 | 91.3 | 51.0 | 43.9 | 94 bp | 2021_145 | ERR11532928 |
| MAAK_03_S8_R1_001 | 234.1 | 90.7 | 53.0 | 31.7 | 74 bp | 2020_185 | ERR11532932 |
| MAAK_03_S13_R1_001 | 10.2 | 69.3 | 53.0 | 28.2 | 70 bp | 2020_143 | ERR11532921 |
| MAAK_03_S13_R2_001 | 10.2 | 68.9 | 54.0 | 28.2 | 70 bp | 2020_143 | ERR11532921 |
| MAIV_24_S5_R1_001 | 229 | 85.8 | 47.0 | 25.1 | 92 bp | 2020_185 | ERR11532950 |
| MAIV_24_S8_R1_001 | 8.5 | 51.3 | 47.0 | 24.5 | 91 bp | 2020_143 | ERR11532922 |
| MAIV_24_S8_R2_001 | 8.5 | 43.8 | 49.0 | 24.5 | 91 bp | 2020_143 | ERR11532922 |
| MAIV_24_S9_R1_001 | 199.6 | 85.0 | 43.0 | 14.4 | 98 bp | 2021_145 | ERR11532951 |
| MAIV_32_S6_R1_001 | 287.3 | 87.1 | 43.0 | 9.1 | 91 bp | 2020_185 | ERR11532953 |
| MAIV_34_S7_R1_001 | 262.9 | 87.8 | 46.0 | 20.9 | 90 bp | 2020_185 | ERR11532955 |
| MAIV_34_S10_R1_001 | 10 | 55.1 | 46.0 | 19.7 | 88 bp | 2020_143 | ERR11532923 |
| MAIV_34_S10_R2_001 | 10 | 48.4 | 48.0 | 19.7 | 88 bp | 2020_143 | ERR11532923 |
| 3655_1_S1_R1_001 | 19.2 | 89.9 | 49.0 | 87.3 | 97 bp | 2020_184 | ERR11547222 |
| 3655_1_S1_R2_001 | 19.2 | 92.8 | 51.0 | 87.3 | 94 bp | 2020_184 | ERR11547222 |
| 3655_2_S2_R1_001 | 16.1 | 90.0 | 49.0 | 91.9 | 98 bp | 2020_184 | ERR11532856 |
| 3655_2_S2_R2_001 | 16.1 | 94.0 | 51.0 | 91.9 | 96 bp | 2020_184 | ERR11532856 |
| HI_LUIN_26_1_S3_R1_001 | 15.7 | 88.7 | 50.0 | 92.3 | 98 bp | 2020_184 | ERR11532858 |
| HI_LUIN_26_1_S3_R2_001 | 15.7 | 93.6 | 51.0 | 92.3 | 96 bp | 2020_184 | ERR11532858 |
| HI_LUIN_26_2_S4_R1_001 | 17.9 | 90.1 | 50.0 | 94.0 | 99 bp | 2020_184 | ERR11532860 |
| HI_LUIN_26_2_S4_R2_001 | 17.9 | 94.4 | 51.0 | 94.0 | 96 bp | 2020_184 | ERR11532860 |
| HI_LUIN_28_1_S5_R1_001 | 17.5 | 90.7 | 49.0 | 92.0 | 98 bp | 2020_184 | ERR11532863 |
| HI_LUIN_28_1_S5_R2_001 | 17.5 | 94.4 | 51.0 | 92.0 | 95 bp | 2020_184 | ERR11532863 |
| HI_LUIN_28_2_S6_R1_001 | 20.3 | 89.0 | 46.0 | 68.0 | 89 bp | 2020_184 | ERR11532864 |
| HI_LUIN_28_2_S6_R2_001 | 20.3 | 92.5 | 48.0 | 68.0 | 87 bp | 2020_184 | ERR11532864 |
| HI_LUIN_33_1_S7_R1_001 | 18.7 | 90.6 | 49.0 | 95.5 | 99 bp | 2020_184 | ERR11532865 |
| HI_LUIN_33_1_S7_R2_001 | 18.7 | 94.9 | 51.0 | 95.5 | 96 bp | 2020_184 | ERR11532865 |
| HI_LUIN_33_2_S8_R1_001 | 17.4 | 89.7 | 50.0 | 93.0 | 98 bp | 2020_184 | ERR11532868 |
| HI_LUIN_33_2_S8_R2_001 | 17.4 | 94.2 | 51.0 | 93.0 | 95 bp | 2020_184 | ERR11532868 |
| HI_MAAK_03_1_S13_R1_001 | 15.8 | 91.9 | 50.0 | 89.1 | 97 bp | 2020_184 | ERR11532888 |
| HI_MAAK_03_1_S13_R2_001 | 15.8 | 94.5 | 51.0 | 89.1 | 94 bp | 2020_184 | ERR11532888 |
| HI_MAAK_03_2_S14_R1_001 | 17.5 | 92.2 | 50.0 | 88.9 | 97 bp | 2020_184 | ERR11532897 |
| HI_MAAK_03_2_S14_R2_001 | 17.5 | 94.7 | 51.0 | 88.9 | 95 bp | 2020_184 | ERR11532897 |
| HI_MAIV_24_1_S9_R1_001 | 21.6 | 87.3 | 45.0 | 51.9 | 88 bp | 2020_184 | ERR11532906 |
| HI_MAIV_24_1_S9_R2_001 | 21.6 | 91.0 | 46.0 | 51.9 | 85 bp | 2020_184 | ERR11532906 |
| HI_MAIV_24_2_S10_R1_001 | 17.8 | 87.9 | 48.0 | 78.4 | 95 bp | 2020_184 | ERR11532913 |
| HI_MAIV_24_2_S10_R2_001 | 17.8 | 91.7 | 49.0 | 78.4 | 93 bp | 2020_184 | ERR11532913 |
| HI_MAIV_32_1_S11_R1_001 | 21.4 | 89.8 | 50.0 | 87.6 | 96 bp | 2020_184 | ERR11532914 |
| HI_MAIV_32_1_S11_R2_001 | 21.4 | 93.8 | 51.0 | 87.6 | 94 bp | 2020_184 | ERR11532914 |
| HI_MAIV_32_2_S12_R1_001 | 22 | 87.9 | 45.0 | 57.0 | 87 bp | 2020_184 | ERR11532917 |
| HI_MAIV_32_2_S12_R2_001 | 22 | 91.6 | 47.0 | 57.0 | 85 bp | 2020_184 | ERR11532917 |

LEGEND SUPPLEMENTAL TABLE S3: Sequencing data per sample and sequence run. Percent duplicates (Dups), GC content (GC), ribosomal RNA (rRNA), mean sequence length, name of sequence run and accession number at the European Nucleotide Archive for the file.

Supplemental Table S4

******

LEGEND SUPPLEMENTAL TABLE S4: Top 50 significant biological process GO terms enriched for upregulated differentially expressed genes (DEGs). “Annotated” denotes the total number (n) of genes present in core genome annotated to each term. “Significant” denotes the number of these genes belonging to the DEGs, while “Expected” gives the expected number of annotated genes present in DEGs (i.e. same proportion as in core genome). Fold enrichment equals “Significant” divided by “Expected”.

Supplemental Table S5

******

LEGEND SUPPLEMENTAL TABLE S5: Top 50 significant biological process GO terms enriched for the downregulated DEGs. “Annotated” denotes the total number (n) of genes present in core genome annotated to each term. “Significant” denotes the number of these genes belonging to the DEGs, while “Expected” gives the expected number of annotated genes present in DEGs (i.e. same proportion as in core genome). Fold enrichment equals “Significant” divided by “Expected”.

Supplemental Figure S1

A)

***
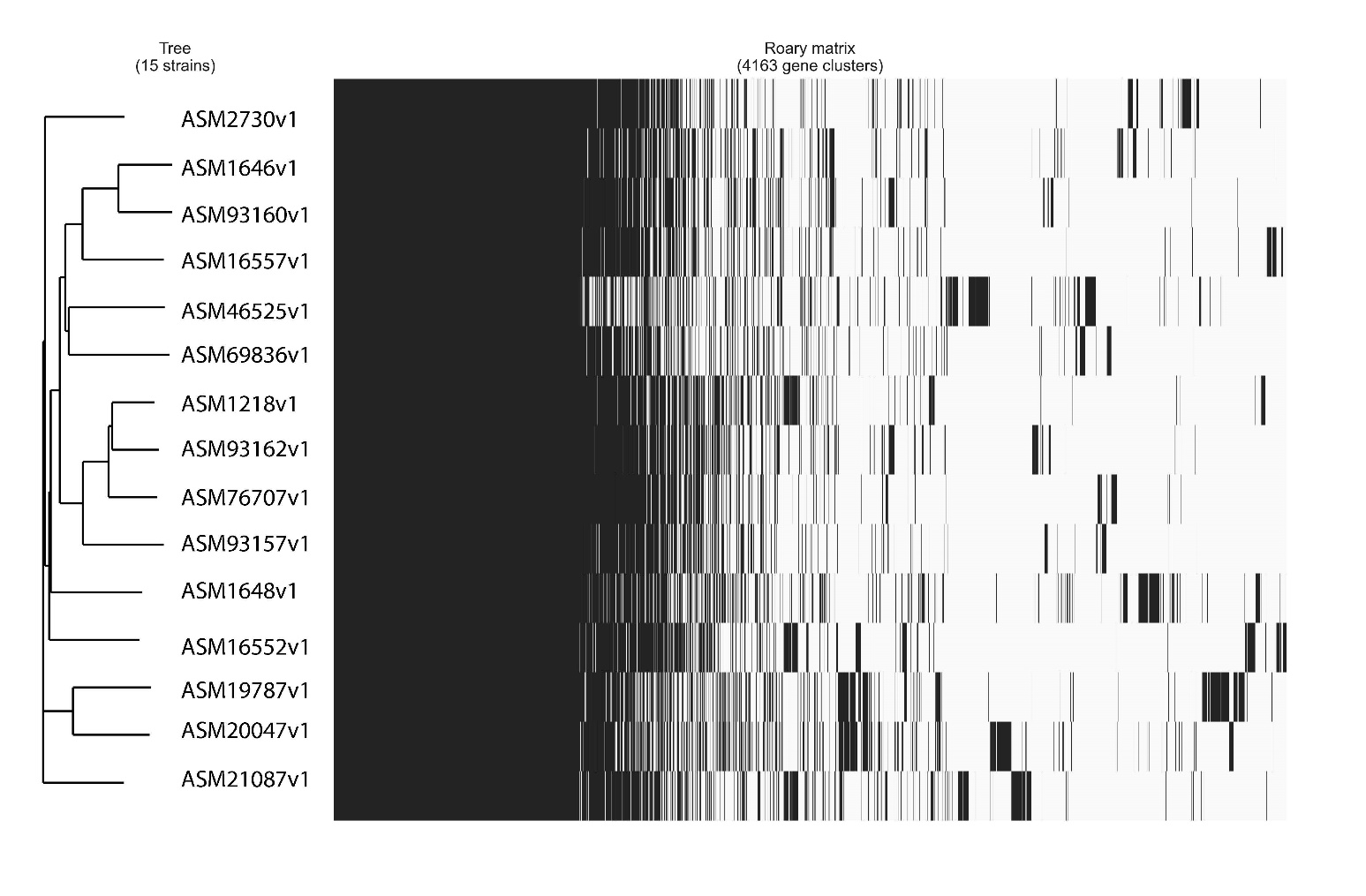
***

B)

***
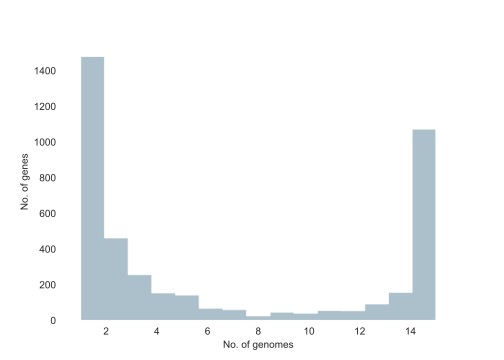
***

C)

***
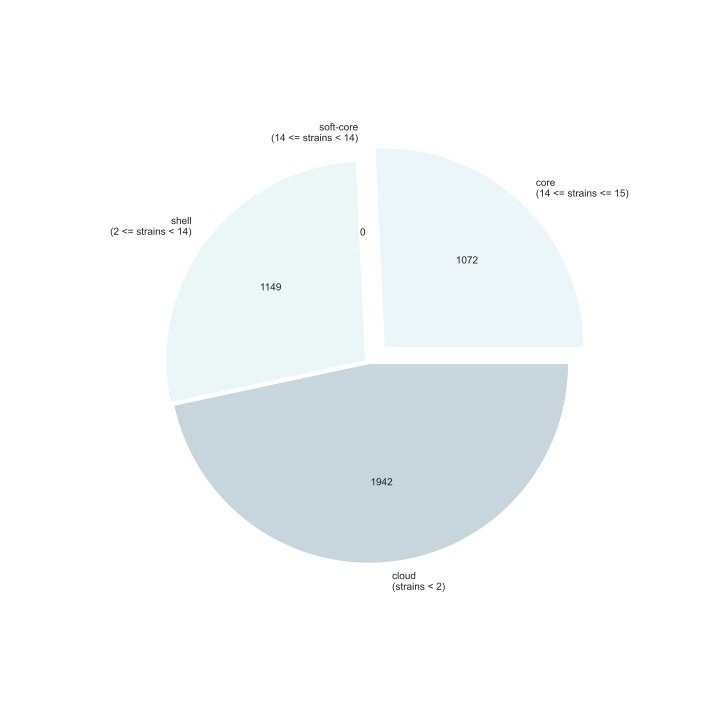
***

LEGEND SUPPLEMENTAL FIGURE S1

Core and pan genomes were created with Roary using 15 reference strains. To illustrate how these strains were related to each other a phylogenetic tree and gene presence/absence matrix were created (A). The strains are here presented by their ASM accession numbers. Full details are listen in Supplemental table S1. A large part of the genes (46.6%) were present in only one reference strain (B). The core genome consisted of 1072 genes, i.e. genes that were present in all 15 strains (C). The shell genome (genes present in 2-14 strains and the cloud genome (genes present in only 1 strain), together with the core genome constitute the pan genome (n=4163). The number inside each segment of the pie chart indicate the number of genes that were included in that segment.

Supplemental Figure S2


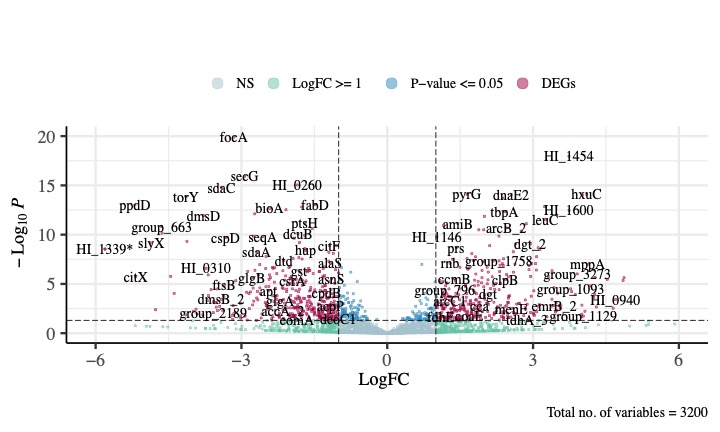


LEGEND SUPPLEMENTAL FIGURE S2: Volcano plot displaying the dispersion of pan genome genes with regards to logFC (x-axis) and p-value (y-axis, log_10_-transformed and inverted adjusted p-values). Names of the most differentially expressed genes shown in graph.

Supplemental Figure S3

LEGEND SUPPLEMENTAL FIGURE S3: Heatmap displaying the normalized counts (variance-stabilized transformed) of the 30 most differentially expressed genes (i.e. genes showing the highest absolute logFC) for each sample. The legend scale indicates the log transformed normalized counts, cropped at -3 and +3.
