# Supplementary material for: In vivo gene expression profile of Haemophilus influenzae during human pneumonia
Source: Microbiol Spectr. 2023 Sep 14;11(5):e01639-23. doi: 10.1128/spectrum.01639-23 (PMC10581191; doi:10.1128/spectrum.01639-23)
Supplement: Supplemental file 2: Bioinformatics commands — Code and commands used for bioinformatic analysis [file spectrum.01639-23-s0003.docx]

***In vivo* gene expression profile of *Haemophilus influenzae* during human pneumonia**

Linnea Polland^1,2^, Hanna Rydén^1,2^, Yi Su^1^, Magnus Paulsson^1,2 #^

Author affiliations

1. Infection medicine, Department of Clinical Sciences Lund, Medical Faculty, Lund University, Lund, Sweden
2. Clinical Microbiology, Office for Medical Services, Region Skåne, Lund, Sweden

**Supplemental file 2: Bioinformatics commands**

###################################################################

##### 1.0 Quality Asessment and trimming of sample sequences

# TrimGalore/0.4.4

# FastQC/0.11.8

# sortmerna/4.3.6

### paired-end reads

#fastqc assessment

fastqc -t 8 -o file_01_R1.fastq.gz

fastqc -t 8 -o file_01_R2.fastq.gz

#trim_galore

trim_galore --stringency 5 --paired --dont_gzip -o file_01_R1.fastq.gz file_01_R2.fastq.gz

#rrna

sortmerna \

--threads 16 \

-ref silva_rrna/silva-bac-16s-id90.fasta \

-ref silva_rrna/silva-bac-23s-id98.fasta \

-ref silva_rrna/silva-euk-18s-id95.fasta \

-ref silva_rrna/silva-euk-28s-id98.fasta \

-reads file_01_R1.fastq.gz

-reads file_01_R2.fastq.gz

### single-end reads

#fastqc assessment

fastqc -t 8 -o file_01_R1.fastq.gz

#trim_galore

trim_galore --stringency 5 --dont_gzip -o file_01_R1.fastq.gz

#rrna

sortmerna \

--threads 16 \

-ref silva_rrna/silva-bac-16s-id90.fasta \

-ref silva_rrna/silva-bac-23s-id98.fasta \

-ref silva_rrna/silva-euk-18s-id95.fasta \

-ref silva_rrna/silva-euk-28s-id98.fasta \

-reads file_01_R1.fastq.gz

###################################################################

##### 2.0 Human Read Removal

# Kraken2/2.1.1

# seqtk/1.2

# bowtie2/2.4.4

# samtools/1.15.1

## 2.1 Clean sequences with kraken2 results

# kraken paired-end reads

kraken2 --use-names \

--threads 64 \

--db ../database/ \

--gzip-compressed \

--report file_01.report.kraken \

--output file_01.kraken \

--paired file_01_R1.fastq.gz file_01_R2.fastq.gz

# kraken single-end reads

kraken2 --use-names \

--threads 64 \

--db ../database/ \

--gzip-compressed \

--report file_01.report.kraken \

--output file_01.kraken \

file_01_R1.fastq.gz

## 2.2 Remove sequences classified as homo sapiens

# extract seq id from kraken files into list

grep -v 'Homo sapiens' file_01.kraken | cut -f2 > list_cleaned_file_01

# seqtk subseq the listed ids from original fastq into new cleaned_fastq files

seqtk subseq file_01_R1.fastq.gz list_cleaned_file_01 | gzip > cleaned/file_01_R1.fastq.gz

## 2.3 Find human reads with Bowtie2

# bowtie2 paired-end

bowtie2 -t -p 16 -x GRCh37/GRCh37 \

-1 file_01_R1.fastq.gz \

-2 file_01_R2.fastq.gz \

-S file_01.sam

# bowtie2 single-end reads

bowtie2 -t -p 32 -x ../bowtie2/GRCh37/GRCh37 \

-U file_01_R1.fastq.gz \

-S file_01_R1.sam

## 2.4 Clean sequences using SAMtools with bowtie2 results

# paired-end

# convert SAM to BAM

samtools view -b file_01.sam -o file_01.bam

# sort in order of names

samtools sort -n file_01.bam -o file_01.bam

samtools fastq -f 0x4 \

-1 file_01_R1.fastq.gz \

-2 file_01_R2.fastq.gz \

-0 /dev/null \

-s /dev/null -n file_01.bam

# single-end reads

# convert SAM to BAM

samtools view -b file_01_R1.sam -o file_01.bam

samtools fastq -f 0x4 \

-0 file_01_R1.fastq.gz \

-s /dev/null -n file_01_R1.bam

###################################################################

##### 3.0 Taxonomy of sample sequences

## 3.1 Make kraken reports for sample

# kraken paired-end reads

kraken2 --use-names \

--threads 64 \

--db ../database/ \

--gzip-compressed \

--report file_01.report.kraken \

--output file_01.kraken \

--paired file_01_R1.fastq.gz file_01_R2.fastq.gz

# kraken single-end reads

kraken2 --use-names \

--threads 64 \

--db ../database/ \

--gzip-compressed \

--report file_01.report.kraken \

--output file_01.kraken \

file_01_R1.fastq.gz

## 3.2 Bracken estimation

python ../miniconda3/envs/kraken/bin/est_abundance.py \

-k database/database100mers.kmer_distrib \

-i file_01.report.kraken \

-o file_01.bracken.species.txt \

-l S

###################################################################

##### 4.0 Core genome and Pan genome creation

# Prokka/1.11

# Roary/15.36.42

# seqtk/1.2

# transeq/EMBOSS:6.6.0.0

## 4.1 Prokka annotation for each of the 15 Hi genomes from ncbi (loop or repeat this for all files)

prokka --kingdom Bacteria \

--outdir prokka_GCA_000027305.1 \

--genus Haemophilus \

--proteins GCF_000027305.1_ASM2730v1_genomic.faa.gz \

--cpus 20 \

--prefix GCF_000027305.1 \

--force GCF_000027305.1_ASM2730v1_genomic.fna.gz

## 4.2 Roary pangenome creation

#using all 15 gff files from prokka

roary -e --mafft -p 20 *.gff

#simplify headers

sed 's/>.*\s\(.*\)/>\1/' pan_genome_reference.fa > pan_genome_reference_simpleheader.fa

## 4.2.1 Extract coregenome

seqtk subseq pan_genome_reference_simpleheader.fa list_coregene > coregenome.fa

## 4.2.2 Roary Plots

python roary_plots.py tree.file gene_presence_absence.csv --format pdf --labels

## 4.3 Annotation of core and pangenome

transeq -sequence coregenome.fa -outseq coregenome.faa -table 11 -trim

transeq -sequence pan_genome_reference_simpleheader.fa -outseq pan_genome_reference_simpleheader.faa -table 11 -trim

###################################################################

##### 5.0 Kallisto pseudo-alignment

# kallisto/0.46.2

#paired-end to pangenome

kallisto quant \

--index idx/HI_pan_kallisto.idx \

--threads 16 \

-o HI_pan \

file_01_R1.fastq.gz \

file_01_R2.fastq.gz

#single-end to pangenome

kallisto quant \

--index idx/HI_pan_kallisto.idx \

--threads 16 \

--single \

--fragment-length 75 \

--sd 20 \

-o file_01_R1.fastq.gz

# use HI_core_kallisto.idx for coregenome pseudoalignment

###################################################################

##### END OF SCRIPT
